# Natural Compounds of Fungal Origin with Antimicrobial Activity—Potential Cosmetics Applications

**DOI:** 10.3390/ph16091200

**Published:** 2023-08-23

**Authors:** Katarzyna Sułkowska-Ziaja, Monika Trepa, Aldona Olechowska-Jarząb, Paweł Nowak, Marek Ziaja, Katarzyna Kała, Bożena Muszyńska

**Affiliations:** 1 Department of Pharmaceutical Botany, Faculty of Pharmacy, Jagiellonian University Medical College, Medyczna 9, 30-688 Kraków, Poland; 2 Department of Pharmaceutical Microbiology, Faculty of Pharmacy, Jagiellonian University Medical College, Medyczna 9 Street, 30-688 Kraków, Poland; 3 Department of Microbiology, University Hospital, ul. Jakubowskiego 2, 30-688 Kraków, Poland; 4 Department of Histology, Faculty of Medicine, Jagiellonian University Medical College, Kopernika 7, 31-034 Kraków, Poland

**Keywords:** antibacterial activity, antifungal activity, antivirus activity, compounds of fungal origin

## Abstract

The phenomenon of drug resistance in micro-organisms necessitates the search for new compounds capable of combating them. Fungi emerge as a promising source of such compounds as they produce a wide range of secondary metabolites with bacteriostatic or fungistatic activity. These compounds can serve as alternatives for commonly used antibiotics. Furthermore, fungi also accumulate compounds with antiviral activity. This review focuses on filamentous fungi and macrofungi as sources of antimicrobial compounds. The article describes both individual isolated compounds and extracts that exhibit antibacterial, antifungal, and antiviral activity. These compounds are produced by the fruiting bodies and mycelium, as well as the biomass of mycelial cultures. Additionally, this review characterizes the chemical compounds extracted from mushrooms used in the realm of cosmetology; specifically, their antimicrobial activity.

## 1. Introduction

In the kingdom of fungi, extensive biosynthetic capabilities are leading to the production of compounds with complex chemical structures that exhibit high biological activity. Operating as saprophytic organisms, fungi are strongly biochemically related to the composition of the substrate on which they reside. This fact largely determines their biosynthetic capabilities [1]. The main groups of biogenetic compounds originating from fungi can be divided into primary and secondary metabolites [2]. Primary metabolites are compounds derived from the primary metabolism of sugars (monosaccharides, disaccharides, polysaccharides, sugar alcohols, and quaternary amine bases). Secondary metabolites are compounds that can be further subdivided into compounds derived from the metabolism of active acetate (polyketides, isoprenoids, and sterols); compounds derived from the metabolism of fatty acids (polyacetylenes); compounds derived biogenetically from shikimic acid (phenols and phenolic acids); compounds formed from the transformation of amino acids (amines, toxic amines, alkaloids, and peptides); and compounds formed from the transformation of aromatic amino acids (ergot alkaloids) [2]. Another division of fungal metabolites includes nitrogenous compounds—including urea, amino acids, peptides, proteins, lectins, amines, alkaloids, indole derivatives, vitamins, purine compounds, isoxazole derivatives, and phenoxazine derivatives—as well as non-nitrogenous compounds such as carbohydrates, lipids, polyacetylenes, polyketides, isoprenoids, sterols, organic acids, and phenolic compounds [2,3].

The phenomenon of drug resistance among micro-organisms necessitates the search for new compounds capable of combating them. Fungi are a promising source of such compounds as they produce a wide range of secondary metabolites exhibiting bacteriostatic or fungistatic activity. These compounds can serve as alternatives to commonly used antibiotics. Furthermore, fungi also accumulate compounds with antiviral activity [4].

The development of new technologies, such as metabolomics, offers great potential for the identification and characterization of new natural antimicrobial compounds in the future. This knowledge may be crucial for the development of future therapeutic strategies. While the majority of natural compounds, including those stemming from fungal origin, do not have sufficient therapeutic effectiveness in monotherapy, their use in combination therapy with traditional antibiotics may contribute to the synergy effect of eliminating side effects and improving pharmacokinetic and pharmacodynamic properties [5].

Progress in fermentation technology, separation methodologies, and techniques for structural determination have rendered other micro-organisms appealing as potential reservoirs of novel bioactive secondary metabolites [6,7].

Among the many compounds that are fungal metabolites, many are used in cosmetology, but may also have important clinical significance if only in terms of increasing drug resistance. This problem also applies to aesthetic medicine. There are many biomaterials used in cosmetology and aesthetic medicine, as well as procedures that pose a potential risk of infection with multidrug-resistant strains. The compounds described here are examples of known natural substances whose application in the context of drug resistance may be important. Currently, researchers are looking for compounds of natural origin whose application could be considered in aspects of global problems. One of these is microbial drug resistance [8,9].

Categorizing antimicrobial compounds extracted from macro- and microfungi can depend on their chemical characteristics, modes of operation, or potential uses. Under the framework of classifying compounds by their chemical structure, we can highlight various antimicrobial agents, such as β-glucans present in macrofungi. Their primary mechanism centers around immune modulation. Additionally, we have the terpenoids, primarily originating from macrofungi, whose effects include the inhibition of microbial enzymes. An essential category comprises peptides, chiefly sourced from microfungi, which exert influence by impacting cell membrane permeability and disrupting cellular processes [10,11,12].

The objective of this review article is to present an updated and comprehensive overview of antimicrobial compounds derived from fungi, highlighting recent advancements in the field. The focus of this review encompassed both filamentous fungi and macromycetes. The paper describes the realm of antibacterial, antifungal, and antiviral compounds produced by both the fruiting bodies and mycelium (in the case of filamentous species) and those accumulated by the biomass of mycelial cultures. In addition, the paper delineates the characteristics of chemical compounds extracted from fungi with applications in cosmetology within the context of their antimicrobial activity. The main question posed in this review paper is as follows: how can fungi serve as a source of antimicrobial compounds to combat drug resistance of micro-organisms and what is the scope of their potential applications, including use in cosmetics?

## 2. Compounds of Fungal Origin with Antimicrobial Activity

### 2.1. Antibacterial Activity of Substances of Fungal Origin

#### 2.1.1. Compounds of Fungal Origin with Antibacterial Activity

Numerous scientific studies indicate the antimicrobial activity of individual compounds and specific extracts obtained from fungal fruiting bodies.

It is believed that the presence of fungal fruiting bodies with such properties is due to defense mechanisms formed by fungi to survive in the environment. As the challenge of bacterial resistance to existing antibiotics grows, a variety of naturally occurring compounds exhibiting antimicrobial activity against pathogenic organisms is garnering increasing attention. Notably, one of the first compounds with antibacterial activity was the antibiotic substance sparassol, which was isolated from *Sparassis crispa* in 1920 [13] (Table 1). Over the following decades, the antibiotic activity of more than 2000 macromycetes species was subsequently validated.

##### Key Antibacterial Compounds of Fungal Origin

Fungi are known for producing a wide variety of compounds endowed with antibacterial activity [6]. These compounds exhibit a dual nature, yielding a broad spectrum of activity while also manifesting selective efficacy against specific bacterial strains. Antibiotics are substances that hinder the growth and division of bacteria. The term “antibiotic” was coined by microbiologist Selman Waksman, who discovered two antibiotics: streptomycin and neomycin [26]. Nowadays, antibiotics encompass a spectrum spanning natural substances, semisynthetic derivatives, and synthetic analogs. These agents selectively target various bacterial structures, leading to either a bactericidal or bacteriostatic effect. Antibiotics are categorized into distinct groups based on factors such as their mode of action, chemical structure, or spectrum of activity. For instance, certain antibiotics inhibit the synthesis of bacterial cell walls (e.g., β-lactams), while others impede protein production (e.g., chloramphenicol, tetracycline), or interfere with bacterial RNA and DNA nucleic acids (e.g., quinolones) [27]. In the early 20th century, small doses of penicillin proved highly effective in controlling a significant proportion of bacterial infections. However, as the use of penicillin increased, its effectiveness waned against infections. This phenomenon, identified as antibiotic resistance, stems from the defense mechanisms micro-organisms develop to counteract antibiotics. It is important to note that antibiotic resistance is not a recent occurrence but rather an outcome of bacteria evolving various mechanisms to protect themselves from harmful substances within their environment. This process allows them to quickly adapt to adverse changes [28]. Filamentous fungi, particularly those belonging to the genera *Penicillium*, *Cephalosporium*, *Aspergillus*, and *Fusidium*, are vital organisms in pharmaceutical biotechnology, particularly in the pharmaceutical industry. These fungi are well-known prolific producers of antibiotics, and alongside actinomycetes, they are recognized as the primary sources of antibiotics [29]. Among the most significant antibiotic classes produced by fungi are penicillins, cephalosporins, fusidans, fusafungin, and fumigacin (helvolic acid).

Penicillin

**Figure 1:**
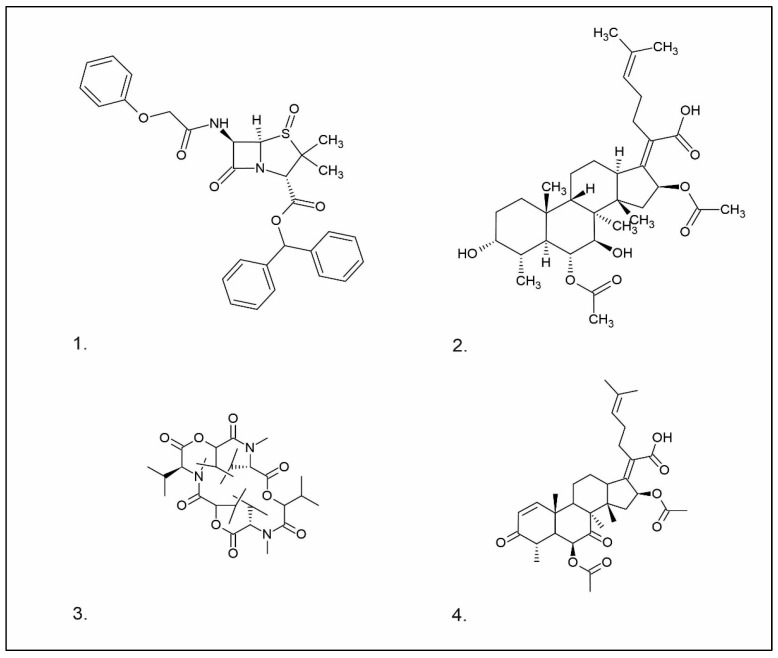
Chemical structures of 1. Penicillin; 2. Cephalosporin; 3. Fusafungine; and 4. helvolic acid.

Penicillins belong to the β-lactam group of antibiotics (Figure 1). In terms of their chemical structure, they contain a thiazolidine ring conjugated with a β-lactam ring. The operational mechanism of penicillins, as well as other β-lactam antibiotics, entails binding to penicillin-binding proteins (PBPs), subsequently obstructing their function. Antibiotics within this grouping exhibit extremely low levels of general and organ toxicity against human cells because they only affect cells where peptidoglycan synthesis occurs. The commercial production of penicillins involves selected species such as *Penicillium chrysogenum*, *Penicillium baculatum*, *Penicillium turbatum*, *Aspergillus persicinum*, *Aspergillus flavus*, *Aspergillus giganteus*, *Aspergillus nidulans*, *Aspergillus oryzae*, and *Aspergillus parasiticus* [30,31]. Penicillins are primarily utilized in medicine to treat bacterial infections. They can be employed for addressing conditions such as impetigo, erysipelas, and acne, thereby contributing to the acceleration of wound healing [31].

Cephalosporin

The precursors of cephalosporins were initially isolated from cultures of *Cephalosporium acremonium* in 1948 by Giuseppe Brotzu [32,33] (Figure 1). The mechanism of action of cephalosporins is analogous to that of β-lactam antibiotics. These compounds find commercial production through strains of *C. acremonium* and *Paecilomyces persicinius*. Cephalosporins can be subdivided into several subgroups based on their chemical structure (P1–P5) [33]. Similar to all β-lactam antibiotics, cephalosporins inhibit the formation of bonds that connect peptidoglycan (murein) subunits, thus preventing the formation of a complete cell wall. They form covalent attachments to the active centers of bacterial enzymes, namely, carboxypeptidase and transpeptidase, leading to the inhibition of their actions. Consequently, they hinder the synthesis of bacterial cell walls. Cephalosporins treat bacterial infections of various origins, including both Gram-positive and Gram-negative bacteria. These antibacterial agents are employed in the treatment of infections caused by pathogens such as *Staphylococcus aureus* and *Escherichia coli*, among others [34]. Cephalosporins can be used to treat skin diseases caused by micro-organisms. In dermatology, they have been applied to address skin conditions such as folliculitis or postoperative infections [35].

Fusidans

One of the most well-recognized fusidans is fusidic acid. Fusidic acid has an inhibitory effect on the protein synthesis of Gram-positive bacteria. Initially isolated in 1962 from *Fusidium coccineum*, fusidic acid was subsequently extracted from *Mucor ramannianus* and *Isaria kogana* [36,37]. Currently, biotechnological methods are obtained to derive fusafungin from species such as *Calcarisporium arbuscula*, *Fusidium coccophilum*, and *Mortierella ramanniana* [36]. Sporting a steroidal configuration, fusidic acid functions as an antibiotic with bacteriostatic properties. Its operational mechanism inhibits the synthesis of bacterial proteins, thereby preventing the growth and multiplication of bacterial cells. Notably, fusidic acid exhibits a narrow spectrum of activity, with a primary focus on Gram-positive bacteria, particularly those that are resistant to penicillin, such as *Staphylococcus* strains. The administration of fusidic acid during treatment might lead to the emergence of resistant strains of *Staphylococcus*. This antibiotic can be used in the form of creams and ointments for the topical treatment of infections such as impetigo, boils, inflammation of sweat glands and hair follicles, atrophy, acne vulgaris, and infections caused by the genus *Staphylococcus* spp. [37,38]. Of significance, fusidic acid can permeate intact skin, with the extent of penetration influenced by factors such as antibiotic exposure duration and skin condition. The biological half-life of fusidic acid is approximately 4–5 h. After being absorbed into the bloodstream, fusidic acid undergoes significant metabolism in the liver. While it is primarily excreted through the bile, a minor portion is eliminated unchanged in the urine [39]. While these compounds are not typically directly utilized in cosmetics due to their medical nature, they do exhibit effectiveness against pathogens responsible for skin diseases, such as *S. aureus* and *Staphylococcus epidermidis* [37].

Fusafungine emerges as a peptide antibiotic that exerts a bacteriostatic effect on numerous pathogenic micro-organisms (Figure 1). In addition to its antibacterial attributes, it also independently demonstrates anti-inflammatory activity. The probable mechanism of action involves enhancing the activity of NK cells, stimulating lymphocytes to produce IL-2, and inhibiting proinflammatory cytokines [40]. Fusafungine has proven efficacy in treating pharyngitis, offering an alternative to systemic antibiotics, steroids, or anti-inflammatory drugs. Sourced from the entomopathogenic fungus *Fusarium lateritium* (Ascomycota), this compound boasts an expansive activity spectrum without inducing bacterial resistance. As an ionophore antibiotic, it amalgamates enniatins and exhibits a unique ability to selectively form complexes with potassium cations, thereby transporting them across the lipid membranes of liposomes [41]. The topical application of fusafungine has been utilized, while its aerosol form has shown promise in treating inflammation of the upper and lower respiratory tract. Clinical trials have confirmed the effectiveness of the aerosolized form of this medication [41].

Fumigacin and helvolic acid (Figure 1) encompass antibiotics and phytotoxic substances produced by fungi belonging to the Ascomycota category, including *Aspergillus fumigatus*, *Cephalosporium caeruleus*, and *Sarocladium oryzae* (known as plant pathogens), alongside the species *Emericellopsis terricola*. Possessing distinctive properties and a varied range of action, fumigacin draws parallels to cephalosporins, especially those within the P1 group [42].

##### Selected Compounds of Fungal Origin from the Group of Isoprenoids, Peptides, and Acetylene Derivatives

Other substances with antibiotic properties, isolated from macrofungi, are compounds classified as isoprenoids, peptides, nucleosides, and acetylene derivatives.

Isoprenoids

Isoprenoid compounds constitute a diverse group of secondary metabolites found in Basidiomycota. These compounds are intrinsically linked to the biogenetic pathway that originates from active acetate and proceeds through mevalonic acid, ultimately leading to the formation of “active isoprene.” Subsequent transformations of the latter undergo a series of transformations, resulting in the production of monoterpenes, sesquiterpenes, diterpenes, triterpenes, tetraterpenes, and steroids [43,44].

Merulidial, which contains an “unsaturated dialdehyde” functional group, emerges from liquid cultures of *Merulius tremellosus* (Table 1). This compound exhibits formidable activity against an array of Gram-positive bacteria, including *Micrococcus roseus*, *Corynebacterium insidiosum*, *Bacillus brevis*, *Bacillus subtilis*, *Streptomyces viridochrontogenes*, *Sarcina lutea*, and *Arthrobacter citreus*, as well as Gram-negative bacteria such as *Proteus vulgaris* [14,45]. Pilatin, a derivative of marasman (Table 1), is isolated from *Flagelloscypha pilati*. It proves effective in inhibiting the growth of Gram-negative bacteria, including *Salmonella typhimurium*, within a concentration range of 5–50 µg/mL [15]. From mycelial cultures of *Pleurotellus hypnophilus*, three metabolites with antibiotic activity have been identified. These include hypnophilin, pleurotellol, and pleurotellic acid (Table 1), all of which are sesquiterpenes derived from hirsutane. The common structural feature shared by all three metabolites is the α-methylenketone moiety [16]. Hypnophilin has been the subject of investigation due to its antimicrobial and antioxidant properties. Its potential utilization in skin care and cosmetics could be attributed to its antioxidant activity, which has the potential to safeguard the skin against oxidative stress and enhance overall skin health. Pleurotellol has been studied for its antibacterial and antifungal properties. In the context of skin care and cosmetics, pleurotellol’s antimicrobial activity could be relevant for formulations targeting skin conditions caused by microbial overgrowth [46]. Lentinellic acid, an iludane-type sesquiterpene (Table 1), exhibits robust antibacterial properties and has been isolated from two species of the genus *Lentinellus*: *Lentinellus omphalodes* and *Lentinellus ursinus*. It exhibits activity against Gram-positive bacteria, including *B. brevis*, *Aerobacter aerogenes*, and *C. insidiosum*, at concentrations ranging from 1 to 5 µL/mL [17]. Sesquiterpenoids with antimicrobial properties could potentially contribute to the development of novel skin care and cosmetic formulations aimed at addressing skin-related concerns caused by micro-organisms. Additionally, their potential antioxidant and anti-inflammatory activities may further enhance their suitability for cosmetic applications, promoting skin health and overall product quality. Lentinellic acid methyl ester also possesses antifungal activity [17]. In the context of skin care and cosmetics, this compound may have potential applications as a preservative in cosmetic formulations to help solve skin problems caused by fungal infections such as athlete’s foot or fungal acne [47].

Sulphurenic acid (Table 1) and eburicoic acid are triterpenes isolated from *Laetiporus sulphureus* [22]. Pleuromutilin, a diterpene compound, was isolated by Kavanagh in 1951 from a saprophytic fungus *Clitopilus passeckerianus* (formerly *Pleurotus passeckerianus*) (Table 1). Pleuromutilin and its derivatives inhibit bacterial protein synthesis by binding to the peptidyltransferase component of the 50S subunit of ribosomes [20]. Striatins A, B, and C are kyatan diterpenes isolated from *Cyathus striatus* (Table 1). These compounds exhibit antibiotic and cytotoxic effects at concentrations of 2 µg/mL. These compounds have been found in both the fruiting bodies and in vitro mycelium of the species. They demonstrate activity against various bacteria, including *A. citreus*, *B. brevis*, *B. subtilis*, *E. coli*, *Leuconostoc mesenteroides*, *Mycobacterium phlei*, *Nocardia brasiliensis*, *P. vulgaris*, *Pseudomonas fluorescens*, *S. lutea*, *S. aureus*, and *Streptomyces viridochromogenes*, along with the fungus *Saccharomyces cerevisiae* and the yeast *Rhodotoula rubra.* In the context of cosmetics and skin care, secondary metabolites such as striatins could have potential applications such as antimicrobial and antioxidant effects [21]. Armillaric acid, isolated from mycelial cultures of *Armillaria mellea*, is a sesquiterpene compound (Table 1) [18]. An aryl ester of this compound, known as melleolide, has exhibited antibacterial activity [48]. *Flammulina velutipes* mycelium has yielded four sesquiterpenes with antibacterial activity: enokipodins A, B, C, and D (Table 1). These compounds exhibit activity against *B. subtilis*, with enokipodins A and C also demonstrating activity against *S. aureus* Enokipodins, with a diverse range of biological properties that could have applications in cosmetics: antioxidant effects, skin brightening, and anti-inflammatory properties [19,49]. From the fruiting bodies of the saprophytic species *Jahnoporus hirtus* (Basidiomycota), a steroid named (24Z)-3,11-dioxolanosta-8,24-dien-26-oic acid has been isolated. This compound displays activity against *Bacillus cereus* and *Enterococcus faecalis* [50]. Ganomycin A and B (Table 1), isolated from *Ganoderma pfeifferi*, exhibit activity against *B. subtilis*, *Micrococcus flavus*, and *S. aureus* [24].

Peptides

**Figure 2:**
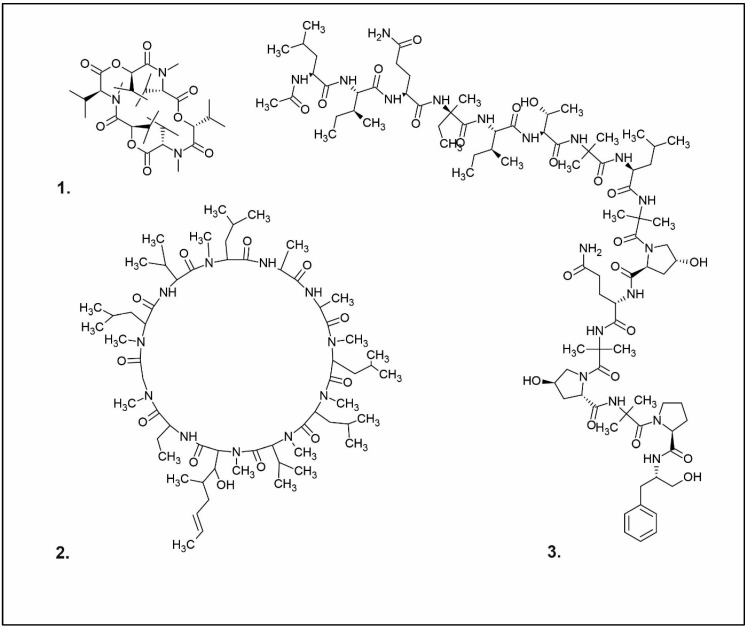
Examples of antibacterial compounds with peptides structure: 1. enniatin; 2. Cyclosporin; and 3. Zervamicin.

One of the peptides produced by fungi is plectasin, isolated from the fruiting bodies of *Pseudoplectania nigrella* (Ascomycota). Plectasin belongs to the defensin group of peptides and carries a cationic character. Comprising 40 amino acids, it exhibits activity against Gram-positive bacteria such as *S. aureus* and *Streptococcus pneumoniae*, primarily affecting the stability of their cell membranes [51]. In vitro, plectasin’s impact on *S. pneumoniae* mirrors that of penicillin and vancomycin. In addition, this peptide targets Gram-positive bacteria of genera such as *Streptococcus* (*S. pneumoniae*, *S. pyogenes*), *Staphylococcus* (*S. aureus*, *S. epidermidis*), *Enterococcus* (*E. faecalis*, *E. faecium*), *Corynebacterium* (*C. diphtheriae*, *C. jeikeium*), and *Bacillus* (*B. cereus*, *B. thuringiensis*) [52]. Zervamicins, a group of peptides with antibacterial activity, is produced by *Emericellopsis salmosynnnemata* (Ascomycota) (Figure 2). These peptides, classified as peptaibols, are linear and characterized by a high content of α,α-dialkyl amino acids, such as α-aminoisobutyric acid [53,54]. Another set of peptaibols includes peptaibol boletusin, peptaibol chrysospermin-3, and peptaibol chrysospermin-5, all extracted from *Boletus* spp. These compounds demonstrate efficacy against *B. subtilis*, *Corynebacterium lilium*, and *S. aureus*. Peptaibol chrysospermin-3 also shows activity against various *Streptococcus* strains [55]. Derived from the fungal fermentation of *Tolypocladium niveum* or *Aspergillus terreus* strains (Figure 2), cyclosporin is a cyclic peptide. The cyclosporin family comprises cyclic peptides with specific amino acids and demonstrates a mild antibiotic effect. However, their primary utility lies in their role as immunosuppressive agents. Cyclosporin A, for instance, is used in medicine to prevent organ rejection in transplant patients and treat autoimmune diseases [56]. Enniatins, cyclic hexapolipeptides, are synthesized by various strains of the *Fusarium* genus within the family *Nectriaceae* (Ascomycota) (Figure 2). In addition to their antibiotic properties, they exhibit insecticidal and anticancer activities [57].

An example of a nucleoside exhibiting antibacterial activity is nebularine, which has been isolated from *Clitocybe nebularis*, a saprotrophic toxic species [58]. Notably, an enzyme with multifaceted attributes, ribonuclease, is sourced from the edible species *Pleurotus sajor-caju*. This enzyme demonstrates antimicrobial, antimitogenic, and antiproliferative effects and exhibits activity against *Pseudomonas aeruginosa* and *S. aureus*, functioning by targeting RNA [59].

Acetylene derivatives

Polyacetylenes stem from gradual desaturation processes involving saturated fatty acids, and they are a common occurrence within the fungal kingdom. Many acetylene derivatives identified in Basidiomycota exhibit antibacterial, cytotoxic, and antifungal activities, such as scorodonin, obtained from *Marasmius scorodonius* (Table 1), and 1-hydroxy-2-nonyn-3-one, extracted from *Ischnoderma benzoinum* [25]. Scorodonin derived from fungi holds potential for diverse cosmetic uses due to its antioxidant, anti-inflammatory, and skin-lightening properties [60]. Aqueveque has isolated two polyacetylenic compounds, namely, hepta-4,6-diyn-3-ol and 7-chloro-hepta-4,6-diyn-3-ol, from *Gymnophilus spectabilis*. These compounds are thought to arise from the desaturation pathway of saturated fatty acids and serve as precursors for the synthesis of polyacetylene compounds in fungi. Their antibacterial activity against Gram-positive and Gram-negative bacteria, as well as their antifungal activity, can be attributed to the presence of unsaturated triple bonds [61].

##### Other Compounds of Fungal Origin with Antibacterial Activity

Pleurotin, a derivative of quinone, along with leucopleurotin and dihydropleurotinic acid, has been isolated from *Pleurotus griseus* (now classified as *Hohenbuehelia grisea*). These compounds exhibit activity against Gram-positive bacteria and specific pathogenic fungi. Pleurotin has shown antimicrobial activity against certain bacteria and fungi. In the context of cosmetics, its antimicrobial properties could be explored for potential use as a natural preservative to prevent microbial growth in cosmetic products [62]. Oxalic acid, isolated from the mycelium of *Lentinus edodes*, shows activity against *B. cereus*, *S. aureus*, and *E. faecalis* [63]. Oxalic acid derived from fungi presents an array of cosmetic uses due to its exfoliating, brightening, antibacterial, and antioxidant properties [64]. Another compound, cloratin A, a benzoic acid derivative, has been isolated from the saprotrophic inedible fungus *Xylaria intracolarata*. This compound displays activity against *E. coli*, *Klebsiella pneumoniae*, *P. aeruginosa*, and *Salmonella enterica*, with particularly potent inhibitory activity observed against *K. pneumoniae*, surpassing the control group [65]. Antibacterial activity is also evident in anthraquinone derivatives such as 6–methylxanthopurpurin-3-O-methyl ether, (1 S, 3 S), austrocortilutein, (1 S, 3 R), austrocortilutein, (1 S, 3 S), austrocortirubin, and torosachryson, isolated from *Cortinarius basirubencens*. Compounds of erythroglaucine and emodin, isolated from other *Cortinarius* species, also demonstrated efficacy against *S. aureus* [66]. A fraction labeled B from *Pycnoporus sanguineus*, mainly composed of loccosee-3-one, exhibited activity against *S*. *aureus* and various strains of *Streptococcus* (A, B, C, and G). Compounds isolated from *G. pfeifferi* showed moderate activity against *E. coli*, *Proteus mirabilis*, and *Serratia marcescens*. Quinoline, isolated from the fungus *Leucopaxillus albissimus*, showed activity against *Achromobacter xyloxidans*, *Acinetobacter baumannii*, *Burkholderia cenocepacia*, *Burkholderia loccose*, *Burkholderia multivorans*, *Cytophaga johnsonae*, and *P. aeruginosa*, with the highest activity observed against *C. johnsonae* [67].

Polysaccharides, such as β-glucans, chitin, and its derivative chitosan, are vital components of the fungal cell wall. Chitosan, featuring amino sugars in its composition, exhibits antibacterial activity. Notably, chitosan is found not only in fungi but also present in the shells of arthropods such as crabs, shrimp, squid, and crayfish [68]. Exhibiting a wide antibacterial activity, chitosan proves effective against certain Gram-negative bacteria, Gram-positive bacteria, and fungi. Specifically, it has shown a higher effect on Gram-positive bacteria, including *Listeria monocytogenes*, *Bacillus megaterium*, *B. cereus*, *S. aureus*, *Lactobacillus plantarum*, *L. brevis*, and *L. bulgaris*. While it does display activity against Gram-negative bacteria, such as *E. coli*, *P. fluorescens*, *S. typhimurium*, and *Vibrio parahaemolyticus*, its potency is comparatively weaker [69,70].

Recent studies indicate that chitosan can be obtained biotechnologically from the cell wall of the filamentous fungus *Rhizopus oryzae*. Its antibacterial properties have been tested against *E. coli*, *K. pneumoniae*, and *S. aureus* [71,72]. A summary of the antibacterial activity of compounds derived from fungi is provided in Table 2.

#### 2.1.2. Extracts of Fungal Origin with Antibacterial Activity

A considerable number of studies have focused on evaluating the antibacterial activity of natural raw materials, often by investigating the analysis of complete extracts. Notably, several types of extracts have been extensively examined, including aqueous, ethanol, methanol, chloroform, dichloromethane, ether, and acetone extracts.

*Ganoderma lucidum* stands as a prominent fungal raw material in East Asian traditional medicine, including TCM [73]. Notably, diverse extracts including aqueous, ethanol, methanol, and acetone have demonstrated comparable efficacy against gentamicin sulfate, an aminoglycoside antibiotic. This effectiveness extends to various bacterial species: *E. coli*, *S. aureus*, *K. pneumoniae*, *B. subtilis*, *S. typhimurium*, and *P. aeruginosa* [74]. Other studies have confirmed that acetone extract of *G. lucidum* exhibits antibacterial activity, mainly against Gram-negative *K. pneumoniae* bacteria. Additionally, a synergistic interaction was observed when combining *G. lucidum* extracts with antimicrobial agents such as ampicillin, cefazolin, oxytetracycline, and chloramphenicol. This synergy was particularly pronounced with cefazolin against *B. subtilis* and *Klebsiella oxytoca* [75]. Conversely, a chloroform extract from the edible mycorrhizal fungus *Hygrophorus agathosmus* exhibited inhibition against various pathogenic bacteria, including *E. coli*, *Enterobacter aerogenes*, *S. typhimurium*, *P. aeruginosa*, *S. aureus*, *S. epidermidis*, and *B. subtilis*. Furthermore, this extract demonstrated inhibitory effects on *Candida albicans* and *S. cerevisiae* [76]. In a similar vein, a dichloromethane extract from *Suillus collitinus* observed activity against Gram-positive bacteria, including *S. epidermidis* and *B. subtilis*. This extract exhibited substantial antibacterial activity, with MIC values of 7.81 µg/mL, surpassing the reference antibiotic streptomycin (MIC = 15.62 µg/mL). For *S. aureus*, MIC values remained equal to those of streptomycin, at 15.62 µg/mL [76]. Finally, the methanolic extract of *Hypholoma fasciculare*, a saprotrophic poisonous fungus, exhibited notable antibacterial activity against Gram-positive bacteria such as *B. cereus*, *B. subtilis*, and *S. aureus* [77]. Turkoglu conducted a study investigating the antibacterial activity of ethanol extracts from *L. sulphureus*. The extract displayed inhibitory activity against the growth of Gram-positive bacteria, including *B. subtilis*, *B. cereus*, *Micrococcus luteus*, and *M. flavus* [78]. Another study analyzed the antibacterial activity of different extracts (chloroform, ethyl acetate, and water) from *Lentinula edodes* fruiting bodies. These extracts showed antibacterial activity against *Streptococcus* spp., *Actinomyces* spp., *Lactobacillus* spp., *Prevotella* spp., and *Porphyromonas* spp., which are known to cause various oral infections. Specifically, chloroform extracts exhibited bactericidal activity against both growing and resting bacterial cells of *Streptococcus mutans* and *Prevotella intermedia*, while the other two extracts exhibited bacteriostatic activity against both growing and resting bacterial cells of *S. mutans* and resting bacterial cells of *P. intermedia* [79]. Furthermore, a low molecular weight fraction study was conducted on an extract of *L. edodes* formulated as a mouthwash and administered to a group of volunteers [80]. Methanolic extract from the mycelium of *Leucopaxillus giganteus*, an inedible saprophytic species, showed antibacterial properties against Gram-positive bacteria in the order of potency: *S. aureus* > *B. cereus* > *B. subtilis*. This study also revealed that diammonium hydrogen phosphate was the preferred nitrogen source for enhancing the production of bioactive compounds inhibiting the growth of Gram-positive bacteria [81]. Studies on methanolic extracts of *Phellinus rimosus* and *Navesporus loccose* demonstrated moderate antibacterial activity against Gram-positive bacteria *B. subtilis* and *S. aureus* [82]. Ethanolic extracts from *Pleurotus ostreatus* and *Meripilus giganteus* exhibited broad-spectrum antibacterial activity, particularly against *S. lutea* [83]. Evaluating extracts from fruiting bodies and mycelial cultures of *Trametes versicolor*, researchers found varying antibacterial activity based on the type of solvent used for the extraction (water, organic solvents, or mixtures). The study revealed significant antibacterial activity against Gram-positive bacteria, with lower activity against Gram-negative bacteria. This effect was attributed to coriolin, a sesquiterpene compound found in *Trametes* (formerly *Coriolus)* spp. (Table 1). Extracts from *Clavariadelphus loccose* and *T. versicolor* have exhibited activity against a range of bacteria, including *E. coli*, *E. aerogenes*, *S. typhimurium*, *S. aureus*, and *B. subtilis* [76]. Aqueous extracts of *Cordyceps sinensis* and *Cordyceps militaris*, which are species that parasitize invertebrates, have demonstrated antibacterial activity against *S. aureus*, probably as a result of an increase in phagocytic macrophage activity and cytokine expression [83]. Ethanol extracts containing polysaccharides from *Grifola loccose* fruiting bodies have been tested against Gram-positive bacteria such as *S. aureus*, *E. faecalis*, *B. cereus*, *L. monocytogenes*, and Gram-negative bacteria such as *E. coli*, *Salmonella enteritidis*, *Shigella sonnei*, and *Yersinia enterocolitica*. The most notable antibacterial activity was observed against *B. cereus* [84]. Acetyl acetate extracts from various species growing in Brazil, including *Phellinus* sp., *Gloeoporus thelephoroides*, *Hexagonia hydnoides*, and *Nothopanus hygrophanus*, demonstrated inhibition of growth against bacteria such as *B. cereus*, *L. monocytogenes*, and *S. aureus* [85]. Aqueous, ethanol, methanol, and xylene extracts of *Agaricus bisporus* and *P. sajor-caju*, both saprophytic edible fungi, have shown antibacterial activity against *E. coli*, *E. aerogenes*, *P. aeruginosa*, and *K. pneumoniae*. Consumption of these fungi may provide natural protection against common pathogenic organisms [86]. Methanolic extracts of *Hydnum repandum*, an edible saprophytic species, have demonstrated activity against the Gram-negative bacteria *P. aeruginosa* [87]. The methanolic extract of the fruiting bodies of *Lepista nuda*, another edible fungus, exhibited antibacterial activity against *E. coli* and *P. aeruginosa* [88]. Dichloromethane extract from *S. collitinus* displayed activity against a range of bacteria, including *E. coli*, *E. aerogenes*, *S. typhimurium*, *S. aureus*, and *S. epidermidis*, *B. subtilis*, as well as *C. albicans* and *S. cerevisiae* [76,89]. Regarding *L. sulphureus*, both ethanolic and aqueous extracts from its fruiting bodies have shown antibacterial effects against various strains, including *B*. *subtilis*, *B*. *cereus*, *M. luteus*, *M. flavus*, and *K*. *pneumoniae*. Among these strains, *M. flavus* exhibited the highest susceptibility, while *K. pneumoniae* showed resistance. Although the efficacy of the active extracts was lower compared to commercial drugs, they still demonstrated potential as antibacterial agents. Furthermore, the aqueous extract of *L. sulphureus* fruiting bodies has shown antibacterial effects against *M. flavus* and *L. monocytogenes* [78,90]. Notably, the extract displayed significant efficacy against *L. monocytogenes*, a strain resistant to streptomycin [90]. A summary of the antibacterial activity of fungal extracts can be found in Table 3.

### 2.2. Antifungal Activity of Substances of Fungal Origin

#### 2.2.1. Compounds of Fungal Origin with Antifungal Activity

Due to concerns over the toxicity of polyene antibiotics and synthetic azole derivatives, researchers have turned to exploring natural compounds with antifungal properties. This pursuit is driven by the increasing resistance of *Candida* species to traditional antifungal drugs, prompting the search for alternative resources. The antifungal activity of substances produced by fungi is attributed to both high-molecular-weight compounds such as proteins and peptides, as well as low-molecular-weight compounds, including terpenes (sesquiterpenes), steroids, and organic acids [9]. Numerous studies conducted in this field involve screening extracts derived from fungal materials [92]. Biforminic acid and biformin, a polyacetylene compound, are examples of substances with antifungal properties produced by the saprophytic fungus *Trichaptum biforme*. These compounds were among the earliest bioactive substances of fungal origin to be identified [93]. Griseofulvin is an antibiotic used for treating fungal infections in both humans and animals. Its mechanism of action involves inhibiting the cell divisions of dermatophytes belonging to the genera *Microsporum*, *Epidermophyton*, and *Trichophyton*. Griseofulvin is produced by various species of the *Penicillium* genus, particularly *Penicillium griseofulvum*, *Penicillium aethiopicum*, *Penicillium janezewski*, and *Penicillium lanosus*. Commercial production of griseofulvin involves biotechnological methods using *Penicillium patulum* [94,95]. During the fermentation processes leading to griseofulvin synthesis, other metabolites are formed, primarily intermediates in the antibiotic’s production, such as dehydrogriseofulvin, griseophenone A, griseoxanthone C, griseophenone Y, and dechlorogriseofulvinic acid. These metabolites might have significant implications in the search for new therapeutic agents. Griseofulvin’s mechanism of action involves inhibiting RNA biosynthesis and chitin synthesis, leading to damage to the fungal cell wall. It is commonly used for treating fungal skin, nail, and hair infections. When administered orally, it is well absorbed from the gastrointestinal tract and reaches peak concentration after about 4 h. The half-life of griseofulvin is 16–20 h, and the recommended dosage for treating superficial infections is typically 250 mg every 6 h. However, it is worth noting that griseofulvin has been discontinued from use [94,95]. Agrocybin, a peptide displaying activity against plant pathogens such as *Mycosphaerella arachidicola* and *Fusarium oxysporum*, was isolated from the fruiting bodies of *Agrocybe cylindracea*, a saprophytic Basidiomycota species [96]. Cordymin, a peptide, functions as an inhibitor of the growth of plant pathogens including *Bipolaris maydis*, *M. arachidicola*, *Rhizoctonia solani*, and *C. albicans* (with IC_50_ values of 50 μM, 10 μM, 80 μM, and 0.75 mM, respectively, for the fungal species). *C. albicans*, a yeast species (Saccharomycetes), is known to cause opportunistic infections in immunocompromised patients, while also constituting the natural flora of the digestive tract in 40–80% of the population. Cordymin was isolated from *C. militaris*, a parasitic species classified under Ascomycota [97]. Cordymin is well-known for its ability to improve the health and look of the skin. Its moisturizing qualities help in retaining skin hydration. This substance efficiently fights against oxidative stress triggered by environmental elements such as UV radiation and pollution. By counteracting damaging free radicals, cordymin helps in thwarting premature skin aging. Alongside its antioxidative attributes, cordymin also demonstrates anti-inflammatory effects and properties that promote skin regeneration [97].

In turn, screening studies aimed at identifying naturally occurring fungicides from fungi have demonstrated the potent antifungal impact of the ethanolic extract derived from the fruiting bodies of *Albatrellus dispansus*. Grifolin, the active compound isolated from these bodies, has been identified as the crucial component. Against *Erysiphe graminis*, *Sclerotinia sclerotiorum*, and *Fusarium graminearum*, it exhibited antifungal activity levels of 86.4% and 80.9%, respectively, at a concentration of 304.9 μM [98,99]. Cloratin A, sourced from *X. intracolarata*, displayed activity against *Aspergillus niger* (with an inhibition zone diameter of 15 mm) and *C. albicans* (with an inhibition zone diameter of 17 mm), comparable to the control substance nystatin, which also had an inhibition zone diameter of 17 mm. The diameter of the inhibition zone (IZD) serves as a reliable indicator of the antifungal activity present in the sample [100]. *Lactarius rufus*, an inedible mycorrhizal fungus, accumulates the sesquiterpene rufuslactone, an isomer of the previously described 3,8-oxa-13-hydroxylactar-6-en-5-lactaric acid γ-lactone from *Lactarius necator*. Rufuslactone exhibits antifungal properties against plant pathogens such as *Alternaria alternata*, *Alternaria brassicae*, *Botrytis cinerea*, and *Fusarium graminearum* [101]. Sesquiterpenoids Enokipodins F, G, and I were isolated from the mycelium of *F. velutipes*, a saprotrophic tree species. These compounds exhibit moderate activity against *A. fumigatus*, a pathogenic species affecting mammals, birds, and insects [49]. The protein isolated from the fruit bodies of *Tricholoma giganteum* exhibits antifungal activity against plant pathogens including *Fusarium oxysporum*, *M. arachidicola*, and *Physalospora piricola*. Furthermore, in the same study, trichogin was confirmed to inhibit HIV-1 reverse transcriptase with an IC_50_ value of 83 nM [102]. Oospolactone, sourced from *Gloeophyllum sepiarium*, possesses antifungal activity against *Alternaria* spp., *Fusarium* spp., *Giberella* spp., *Penicillium* spp., and *Aspergillus* spp. [103]. Lentin, isolated from *L. edodes*, also exhibits antifungal activity against *M. arachidicola* [104]. Compounds characterized by the acylcyclopentenodione structure, identified as chrysotriones A and B from *Hygrophorus chrysodon*, an edible saprophytic species, exhibit activity against *Fusarium verticillioides* [105]. Three sterol-structured compounds and five terpene-structured compounds were isolated from the fruiting bodies of *Ganoderma annulare*: 5-ergosta-7-en-3-ol; 5-ergosta-7,22-dien-3-ol; 5,8-epidioxy-5-ergost-6,22-dien-3-ol; and aplplanoxidic acids A, C, F, G, and H. All these compounds were subjected to antifungal activity testing against the dermatophytes *Microsporum canis* and *Trichophyton mentagrophytes*. Applanoxidic acid A displayed the highest level of activity [106]. A summary of the antifungal activity of fungal compounds is presented in Table 4.

#### 2.2.2. Extracts of Fungal Origin with Antifungal Activity

The antifungal activity of both aqueous and ethanol extracts from *A. bisporus* was determined against *A. flavus* [111]. Ethanolic extracts and the protein fraction obtained from the mycelium of *Ophiocordyceps sobolifera* displayed potent antifungal effects against both pathogenic and saprophytic fungi, including *C. albicans* [112]. Methanolic extracts of *A. bisporus*, *Agaricus bitorquis*, and *Agaricus sylvicola* demonstrated antifungal activity against *C. albicans* and *Candida tropicalis* [113]. The chloroform extract of *H. agathosmus* exhibited antifungal activity against *S. cerevisiae* [76]. Additionally, the dichloromethane extract from *S. collitinus* showed activity against both *S. cerevisiae* and *C. albicans* [76]. Numerous fungal fruiting body extracts have been tested for their antifungal activity against *C. albicans* strains. Notably, the ethanolic extract derived from *L. sulphureus* fruiting bodies exhibited significant activity, with an inhibition zone diameter (IZD) measuring 21 ± 1 mm. This result surpassed the positive control, nystatin, which had an IZD of 19 mm [78].

Furthermore, the methanolic extract from *Lactarius camphoratus*, chloroform extract from *L. edodes*, and methanolic extract from *Lepista nuda* also exhibited activity against *C*. *albicans* [87]. Studies have revealed that extracts from *G. lucidum* inhibit the growth of micro-organisms responsible for surface mycoses caused by *Pityrosporum ovale*, *S*. *epidermidis*, and *Propionibacterium acnes* [106,110].

An aqueous extract sourced from *L*. *sulphureus* demonstrated potent antifungal activity, with ketoconazole serving as the benchmark antifungal drug. Although the *L. sulphureus* extract displayed a slightly milder effect in comparison to ketoconazole, it still held promising potential as an antifungal agent. Furthermore, the aqueous–ethanol extract extracted from *L. sulphureus* exhibited antifungal activity targeting a range of fungi, including *A. niger*, *B. cinerea*, *F. oxysporum*, *Penicillium gladioli*, and *Sclerotinia sclerotiorum* [114,115].

*T. versicolor* extracts exhibit not only antifungal activity but also antifungal activity. In a cross-sectional study that evaluated the antifungal activity of various Basidiomycota representatives, the methanolic extract derived from *T. versicolor* showed activity against *A. fumigatus*. However, no discernible activity against *C. albicans* was observed [91]. A summary of the antifungal activity of fungal extracts is provided in Table 5.

### 2.3. Extracts and Chemical Compounds of Fungal Origin with Antiviral Activity

The antiviral mechanisms of fungal-derived substances often involve blocking viral enzymes, disrupting nucleic acid synthesis, or indirectly boosting the immunostimulatory effects. While numerous chemical compounds with proven antiviral activity are registered drugs, ongoing intensive research aims to search for substances of natural origin, including those of fungal origin. The scientific literature broadly describes the antiviral effect of both fruiting body extracts and single, isolated compounds [117]. For instance, triterpenes such as ganoderiol, ganodermanontriol (Table 1), and ganodermic acid derived from *G. lucidum* exhibit activity against HIV-1 [110]. Similarly, ganodermadiol, lucidadiol, and lucidumol B obtained from *Ganoderma pfeiferi* demonstrate effectiveness against the influenza A virus [118]. Ganodermadiol also combats the herpes virus HSV-1 [110]. Phenolic compounds sourced from *Inonotus hispidus* exhibit activity against influenza viruses of types A and B [119]. Among the macromolecular compounds with antiviral activity isolated from fungi, the most noteworthy is the PSK complex (Krestin). This polysaccharide peptide, derived from the mycelium of *T. versicolor*, boasts anticancer and immunostimulatory properties. Scientific studies have confirmed the antiviral activity of PSK against cytomegalovirus and its ability to inhibit HIV replication [120].

In the study of natural substances, special attention has been given to the analysis of aqueous extracts. This is attributed to the logistical challenges and potential hazards associated with the utilization of organic solvents as extraction agents for raw materials. Compounds found within the fruiting bodies of species such as *G. pfeifferi*, *Rozites caperata*, and *Agaricus brasiliensis* have exhibited activity against herpes viruses. Notably, sulfated polysaccharides from *A. brasiliensis*, RC28 proteoglycan from *R. caperata*, and triterpenoids from *G. pfeifferi* (present in aqueous extracts) exhibit noteworthy antiviral potential. These compounds hold the ability to effectively counteract various stages of herpes virus replication [118,121,122].

Aqueous extracts containing polysaccharides and ethanol extracts sourced from *Pleurotus pulmonarius* fruiting bodies have demonstrated antiviral activity against the influenza A (H1N1pdm) virus [123]. Similarly, the acidic polysaccharide fraction obtained from *C. militaris* fruiting bodies has shown identical antiviral activity against the influenza A (H1N1) virus [124].

Aqueous–methanol extracts derived from the fruiting bodies of *L*. *sulphureus* have demonstrated inhibitory effects on HIV reverse transcriptase. This enzyme plays a crucial role in the transcription process, and its inhibition leads to the suppression of virus replication. The observed antiviral activity within the tested extracts is believed to be influenced by the presence of immunomodulatory polysaccharides [125]. Polysaccharides from *A. brasiliensis* show antiviral activity against poliovirus type (PV-1) [126]. In the case of polysaccharides EP-AV1 and EP-AV2 sourced from an aqueous extract of the fruiting body of *Porodaedalea pini* (also known as *Phellinus pini*), their presence inhibits plaque formation in Vero cells induced by herpes simplex virus 1 (HSV-1) and by Coxsackie virus B3 (CVB3) in HeLa cells. These polysaccharides have been demonstrated to affect the initial stage of virus replication [127].

Polyphenols were isolated from the ethanol extract of the fruiting bodies of *Phellinus baumii*. Through spectroscopic techniques, compounds including hispidin, hypholomine B, inoscavin A, davallialactone, and pelligridin D were identified. These compounds demonstrated inhibitory effects on the neuraminidase activity, an enzyme specific to the H1N1, H5N1, and H3N2 strains of the influenza virus. Additionally, they exhibited a reduction in the virus-induced cytopathic effect (CPE). Neuraminidase serves as an enzyme that allows viruses to exit cells by breaking down the cell membrane of an infected cell. It also plays a role in facilitating virus attachment to cell membranes, aiding their entry into the cell due to its high affinity for the sialic acid of membrane receptors [128]. Laccase isolated from *Pleurotus ostreatus* and tyrosinase from *A. bisporus* show activity against HCV. Laccase from *P. ostreatus* has been shown to block viral entry and replication into PBMC and HepG2 cells, while tyrosinase from *A. bisporus* inhibits viral replication into replicon-containing Huh-5-2 cells [129]. Another noteworthy species that show significant antiviral activity is *Grifola frondosa*. The main active compound is β-glucan (GF-D). It has been shown that a combination of GF-D with IFN human interferon α–2b could potentially offer effective therapy against chronic HBV infections [130,131]. In 2018, structural identification of lentinan from *L. edodes* mycelium LNT-1 was conducted, followed by an investigation of its antiviral activity against hematopoietic necrosis virus (IHNV) [132]. Notably, its immunostimulatory activity was also demonstrated. As proven, the innate immune response is a critical factor in the course of COVID-19 disease. COVID-19 patients show high titers of inflammatory cytokines, so the effect of LNT-1 on SARS-CoV-2 should be considered [133].

A potential candidate in the battle against SARS-CoV-2 is *Inonotus obliquus*, commonly known as the chaga fungus, which possesses a robust enzyme system and defense mechanism due to its parasitic lifestyle [133]. SARS-CoV-2, the virus responsible for COVID-19, primarily targets the human respiratory system and other vital organs. Currently, no specific treatment for SARS-CoV-2 infection exists, although certain drugs have displayed potential efficacy in inhibiting the virus. Natural substances, including fungi, have exhibited potent antiviral and anti-inflammatory effects positioning them as promising candidates for effective COVID-19 treatments [133]. *I. obliquus*, commonly found in Asia, Europe, and North America, serves as a widely utilized natural resource for various ailments. A specific polysaccharide fraction derived from *I. obliquus*, named IOP, has shown the ability to inhibit the production of NO and similar cytokines associated with COVID-19 [133]. COVID-19 patients often experience inflammatory responses, resulting in elevated plasma levels of cytokines and leukocytes. Since IOPs have shown promising results in treating various viral diseases, their potential effect on COVID-19 infection holds considerable promise. Furthermore, an aqueous extract of *I. obliquus* has demonstrated virucidal activity against the hepatitis C virus, remarkably reducing its infectivity by 100-fold within a span of 10 min [134,135]. A summary of the antiviral activity of compounds and fungal extracts is provided in Table 6.

## 3. Cosmetic Applications of Antimicrobial Fungal-Derived Compounds

**Figure 3:**
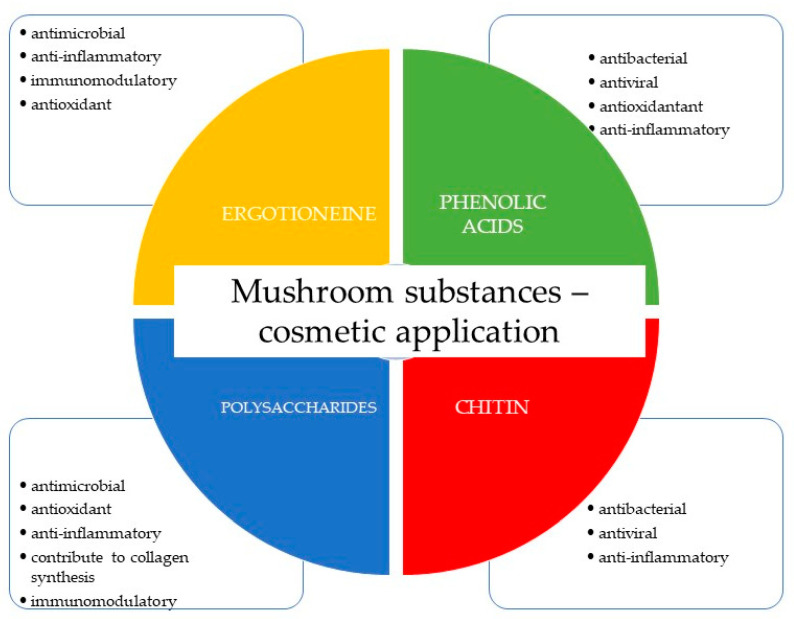
Cosmetic applications of ingredients of mushroom origin are the most used.

Antimicrobial fungal-derived compounds have gained significant attention in various fields, including cosmetics, due to their potential benefits in preventing and treating microbial infections. These compounds offer several applications within cosmetic formulations, particularly in skin care and hair care products (Figure 3) [10,11,12,138,139].

The antimicrobial properties of mushrooms have been extensively explored in recent studies, revealing their potential as valuable sources of natural compounds with antibacterial and antioxidant activities. In the study by Eiamthaworn et al., focused on *Cordyceps militaris* extracts and determining the biological activity of extracts against skin pathogenic bacteria, results demonstrate that *Cordyceps militaris* extracts exhibit significant antibacterial activity against various skin pathogens. This finding underscores the potential of mushrooms as a natural source of antimicrobial agents, holding promise for the development of therapeutic solutions targeting skin infections [140]. In turn, Taofiq et al. describe the development of mushroom-based cosmeceutical formulations. These formulations are designed to possess not only antimicrobial properties but also anti-inflammatory, antityrosinase, and antioxidant activities. The study reveals the multifunctional nature of mushroom compounds, highlighting their potential to address multiple skin-related concerns simultaneously. This research supports the notion that mushrooms can serve as versatile ingredients in skin care products, offering holistic benefits beyond conventional antibacterial effects. The studies suggest that mushroom-derived compounds can contribute to the creation of effective solutions for skin care, particularly in combatting skin infections, reducing inflammation, and providing antioxidant support [139].

### 3.1. Potential Practical Applications

One of the foremost practical applications within the realm of mushroom-derived bioactive compounds lies in their preservative efficacy. Fungal metabolites bearing antimicrobial attributes offer a compelling natural substitute for conventional synthetic preservatives, thereby augmenting the longevity of cosmetic formulations [141].

Furthermore, the utility of mushroom metabolites extends to formulations tailored for acne-prone or sensitive cutaneous conditions. Their antimicrobial prowess can be harnessed to effectively modulate the proliferation of bacteria associated with acne pathology.

Moreover, the strategic integration of fungal metabolites with antimicrobial characteristics into sun protection formulations represents an additional avenue of application. Such products offer a comprehensive approach to skin well-being, amalgamating solar safeguarding, cutaneous hydration preservation, and antimicrobial properties [142].

### 3.2. Microbiological Factors Causing Skin Diseases

Numerous cutaneous disorders, such as acne and fungal infections, stem from pathogenic micro-organisms. Mushroom-derived metabolites possessing potent antibacterial and antifungal attributes hold promising potential in counteracting these infections. Through their incorporation into topical formulations, the capacity arises to selectively target and eradicate detrimental micro-organisms, concurrently fostering skin health. In the realm of bacterial-induced skin maladies, exemplars such as *S. aureus* prevail. This species is a prevalent instigator of conditions ranging from boils and impetigo to cellulitis, and even more severe manifestations such as methicillin-resistant *S. aureus* (MRSA) infections. Research endeavors are oriented towards comprehending the virulence determinants and mechanisms that facilitate *S. aureus*’ cutaneous colonization and invasiveness, with the aim of developing efficacious antibiotic interventions [143]. Additionally, *Streptococcus pyogenes*, recognized as Group A *Streptococcus* (GAS), emerges as a causative agent for afflictions including impetigo, erysipelas, and cellulitis, warranting meticulous scrutiny to unravel its pathogenic intricacies [144]. Similarly, *P. acnes* assumes prominence as the causal agent of acne vulgaris, a prevalent dermatological concern. Scientific inquiry has delved into deciphering the role of *P. acnes* in acne genesis, its interplay with the immune system, and potential therapeutic modalities targeting this bacterium [145]. Among dermatophytes that assail the integument, noteworthy is the prevalence of *Trichophyton* species, attributed to a spectrum of fungal skin infections spanning tinea corporis, tinea pedis (athlete’s foot), and tinea cruris (jock itch). Another cluster of fungi, encompassing *Microsporum* and *Epidermophyton*, contributes to parallel conditions such as athlete’s foot. Comprehensively exploring mushroom-derived metabolites endowed with potent antibacterial and antifungal characteristics, with the goal of addressing this gamut of micro-organisms, underscores an auspicious avenue in advancing therapeutic approaches for diverse cutaneous infections [146].

### 3.3. Selected Compounds of Fungal Origin with Cosmetic Properties

Ergothioneine, an exogenous amino acid derived from thiourea-histidine, emerges as a notable example. This compound boasts high solubility in water and stability at high temperatures. The primary source of ergothioneine lies within macrofungi. Its ability to chelate metal ions aids in preventing the buildup of reactive oxygen species in the body. Furthermore, ergothioneine exhibits antimicrobial and anti-inflammatory properties while contributing to the regulation of immune system function [12,138]. Ergothioneine, derived from fungi, has found extensive applications in cosmetic products. It offers protective properties against mitochondrial DNA damage in fibroblasts and keratinocytes, thereby safeguarding the skin against the harmful effects of free radicals. Its principal application resides in antiaging preparations. In the realm of cosmetology, ergothioneine extracted from *A. bisporus*, *G. frondosa*, and *L. edodes* stands as a widely employed resource [10].

Chitin forms the cell wall of mushrooms, comprising a complex structure of poly-N-acetyl-1,4-D-glucosamine subunits linked by β-(1→4) glycosidic bonds. This compound exhibits insolubility in water while displaying hygroscopic and sorptive characteristics. Remarkably, chitin showcases properties including anti-inflammatory, antiviral, and antibacterial properties [147]. Chitin finds application in cosmetic products, mainly prized for its moisturizing effects. It offers antiaging, firming, and regenerative benefits, reinstating skin elasticity, delivering antioxidant effects, and stimulating collagen synthesis. Its presence is particularly prominent in formulations tailored towards mature and dry skin, effectively maintaining appropriate hydration levels [148].

Chitosan, a deacetylated derivative of chitin, exhibits an affinity for keratin and can create bonds with amino acids. Even at low concentrations, it can create an elastic film that is resistant to moisture. These traits render chitosan suitable for various products, including hair care products [149]. Within both filamentous and macromycetes fungi, polysaccharides are prevalent, and their biological activity is determined by their chemical structures. Predominantly represented by glucans, fungal polysaccharides can also possess galactan or mannans structures. The type of glycosidic bond (α or β) and the arrangement of the polysaccharide molecule significantly impact their biological activity. Of particular note are β-glucans, which exhibit primary biological activity. These polysaccharides often possess substantial molecular weights ranging from 100 to 1000 kDa. The most active polysaccharides tend to sport a linear structure, devoid of long side chains, facilitating enhanced solubility and assimilation. The primary chain typically contains β-(1→3) bonds, with branching occurring through β-(1→6) bonds [147]. Some polysaccharides might even incorporate an additional peptide fragment. Higher fungi-derived polysaccharides have primarily been employed as immunostimulants in the noninvasive treatment of cancer, with lentinan serving as a prime example. These polysaccharides also exhibit diverse effects encompassing antiviral, antibacterial, antiparasitic, anti-inflammatory, hypotensive, hypoglycemic, and vasoprotective effects [69]. Mushroom-derived polysaccharides in cosmetic preparations are utilized for their antioxidant properties. These compounds contribute to collagen synthesis, protect skin cells from UV radiation, demonstrate anti-inflammatory and antimicrobial effects, and are employed in cosmetics for their moisturizing effects [72].

Phenolic acids, notably gallic acid, not only exhibit antioxidant properties but also wield anti-inflammatory, antibacterial, and antiviral properties. Gallic acid finds application in combating pigmentary disorders, which stand among the most common dermatological conditions [150].

Vitamin E, a term often used to depict a collection of closely related compounds named tocopherols and tocotrienols, possesses a shared structure featuring a chromanol and an isoprene side chain. This vitamin is recognized for its capability to scavenge loose radicals, which, in turn, is thought to safeguard against degenerative ailments such as cancer and cardiovascular disorders. Moreover, vitamin E showcases prowess in combatting diverse micro-organisms and impeding their growth. This valuable attribute of vitamin E contributes to its potential role in bolstering immune system well-being and fortifying the body’s resistance against infections. In the realm of cosmetology, vitamin E holds significant sway due to its favorable impacts on the skin, encompassing antioxidant, moisturizing, regenerative, UV radiation shielding, anti-inflammatory, and anti-aging properties [151]. As a potent antioxidant, vitamin E diligently counters free radicals that pose harm to skin cells and expedite the aging process. It erects a safeguarding barrier to stave off moisture loss, thereby preserving skin suppleness and thwarting desiccation. The vitamin aids in the regeneration of compromised skin cells and exhibits particular efficacy in reducing the visibility of scars. While not a replacement for sunscreen, vitamin E furnishes a measure of defense against UV-induced harm. Scientifically established as a formidable antioxidant with anti-inflammatory attributes, vitamin E further amplifies its merits. Furthermore, the vitamin’s adeptness in fending off oxidative stress and facilitating skin restoration enhances its prowess in the battle against aging. Mushrooms stand as a natural wellspring of vitamin E. Among the species that have been scrutinized for their tocopherol and tocotrienol content, *Lentinula edodes*, *Agaricus bisporus*, *Pleurotus ostreatus*, and *Grifola frondosa* [152].

Inhibiting tyrosinase, the enzyme responsible for melanin formation, is a pivotal strategy for skin brightening. Many substances used for skin brightening modify tyrosinase activity [139]. Among compounds derived from fungi boasting skin-whitening qualities are kojic acid, azelaic acid, and 3,4-dihydroxybenzaldehyde [153]. Kojic acid is a crucial inhibitor of melanin biosynthesis, mirroring the action of hydroquinone. It functions by obstructing tyrosinase activity while additionally offering antimicrobial and antioxidant properties. This compound is primarily produced by fungal species belonging to the *Aspergillus* and *Penicillium* genera [154]. Azelaic acid stands as another example of a compound influencing tyrosinase activity. It is produced, among other sources, by the species *P. ovale* (Basidiomycota). Azelaic acid’s effectiveness against hyperpigmentation has been substantiated, even surpassing that of hydroquinone. In addition, it is characterized by keratolytic, anti-inflammatory, and antibacterial effects [139].

## 4. Conclusions

The escalating prevalence of infectious diseases has escalated into a global concern, primarily attributed to the increasing resistance that micro-organisms and viruses have developed against conventional antimicrobial drugs utilized for both therapeutic and preventive purposes. In response to this critical challenge, there has been a notable surge in the exploration of natural sources endowed with potent antimicrobial properties. Within the scientific literature, fungi emerge as compelling candidates, showcasing robust antimicrobial potential that spans across a diverse spectrum, encompassing Gram-positive and Gram-negative bacterial strains, fungal pathogens, and even viruses. This burgeoning interest in fungi’s antimicrobial capabilities is particularly intriguing in the context of the cosmetics industry, which is actively harnessing these therapeutic attributes to elevate the quality and efficacy of its products. A comprehensive assessment of conducted studies underscores the remarkable presence of substances with substantial antimicrobial activity within mushrooms, emblematic representatives of the fungal kingdom. These bioactive compounds hold immense promise for diverse applications, particularly in the formulation of skin care products tailored to address a range of persistent skin conditions. Furthermore, the therapeutic potential of these fungal substances extends beyond cosmetics, demonstrating effectiveness in the treatment of various dermatological diseases. This signifies a significant stride towards merging the realms of natural antimicrobial agents derived from fungi with the intricacies of dermatological care.

## Figures and Tables

**Table 1:** Chemical structures of examples of compounds with antibacterial activity of fungal origin.

Group of Compounds	Compound	Species	Chemical Formula	Reference
Benzoic acid derivative	Sparassol	*Sparassis crispa*	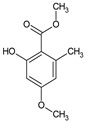	[13]
Sesquiterpenes (C15)	Merulidial	*Merulius tremellosus*	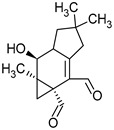	[14]
	Pilatin	*Flagelloscypha pilatii*	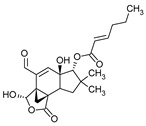	[15]
	Hypnophilin	*Pleurotellus hypnophilus*	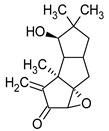	[16]
	Pleurotellol	*Pleurotellus hypnophilus*	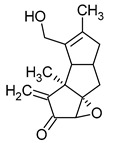	[16]
	Lentinellic acid	*Lentinellus omphalodes* *Lentinellus ursinus*	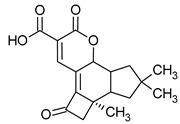	[17]
	Armillaric acid	*Armillaria mellea*	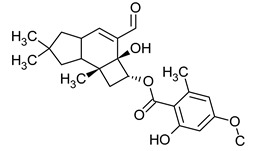	[18]
	Enokipodin A	*Flammulina velutipes*	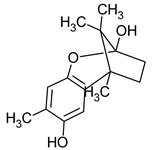	[19]
	Coriolin	*Coriolus consors*	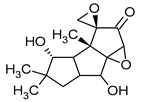	[3]
Diterpenes (C20)	Pleuromutilin	*Clitopilus passeckerianus*	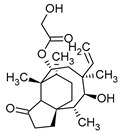	[20]
	Striatin A	*Cyathus striatus*	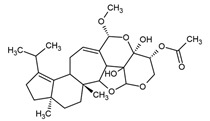	[21]
Triterpenes (C30)	Sulphurenic acid	*Laetiporus sulphureus*	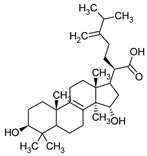	[22]
	Ganodermanontriol	*Ganoderma lucidum*	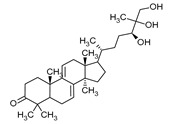	[23]
Meroterpenoids (C40)	Ganomycin A	*Ganoderma pfeiferii*	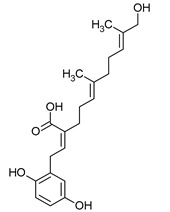	[24]
Acetylene derivatives	Scorodonin	*Marasmius scorodonius*	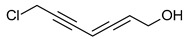	[25]
Sterols	Ganoderiol	*Ganoderma lucidum*	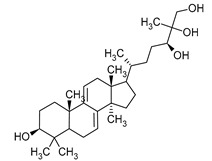	[23]

**Table 2:** Summary of antibacterial activity of compounds of fungal origin.

Species	Extract	Bacteria	References
*Aspergillus giganteus* *Aspergillus nidulans* *Aspergillus oryzae* *Aspergillus parasiticus* *Aspergillus persicinum* *Aspergillus flavus* *Penicilium baculatum* *Penicilium chrysogenum* *Penicillium turbatum* *Penicillum chrysogenum*	Penicillins	Gram-positive bacteria *Diplococcus* spp. *Enterococcus* spp. *Staphylococcus* spp. *Streptococcus* spp. Gram-negative bacterial *Clostridium* spp. *Enterobacteriaceae* spp.	[30,31]
*Cephalosporium acremonium*	Cephalosporins	Gram-positive bacteria: *Diplococcus* spp. *Enterococcus* spp. *Staphylococcus* spp. *Streptococcus* spp. Gram-negative bacteria: *Clostridium* spp. *Enterobacteriaceae* spp.	[32,33,34,36,38,39]
*Calcarisporium arbuscula* *Fusidium coccineum* *Isaria kogana* *Mucor ramannianus*	Fusidans	Gram-positive bacteria	[36,37,38,39]
*Fusarium lateritium*	Fusafungine	*Streptococcus pyogenes* *Streptococcus pneumoniae* *Staphylococcus epidermidis* *Moraxella catarrhalis* *Legionella pneumophila* *Mycoplasma pneumoniae*	[40,41]
*Aspergillus fumigatus* *Cephalosporium caeruleus* *Emericellopsis terricola* *Sarocladium oryzae*	Fumigacin (helvolic acid)	Gram-negative bacteria	[42]
*Merulius tremellosus*	Merulidial	Gram-positive bacteria: *Arthrobacter citreus* *Bacillus brevis* *Bacillus subtilis* *Corynebacterium insidiosum* *Sarcina lutea* *Streptomyces viridochrontogenes* Gram-negative bacteria: *Proteus vulgaris*	[14]
*Flagelloscypha pilati*	Pilatin	*Salmonella typhimurum*	[15]
*Pleurotellus hypnophillus*	Hypnophilin Pleurotellol Pleurotellic acid	*Bacillus brevis* *Salmonella typhimurium*	[16]
*Lentinellus omphalodes* *Lentinellus ursinus*	Lentinellic acid	*Bacillus brevis* *Aerobacter aerogenes* *Corynebacterium insidosum*	[17]
*Laetiporus sulphureus*	Sulphurenic acid Eburicoic acid	Gram-positive bacteria	[22]
*Clitopilus passeckerianus*	Pleuromutilin	*Mycoplasma* spp. *Brachyspira hyodysenteriae* *Brachyspira pilosicoli*	[20]
*Cyathus striatus*	Striatins A, B, C	*Arthrobacter citreus* *Bacillus brevis* *Bacillus subtilis* *Escherichia coli* *Leuconostoc mesenteroides* *Mycobacterium phlei* *Nocardia brasiliensis* *Proteus vulgaris* *Pseudomonas fluorescens* *Sarcina lutea* *Staphylococcus aureus* *Streptomyces viridochromogenes*	[21]
*Armillaria mellea*	Armillaric acid	Gram-positive bacteria	[18]
*Flammulina velutipes*	Enokipodin	*Bacillus subtilis* *Staphylococcus aureus*	[49]
*Jahnoporus hirtus*	(24Z)-3,11-Dioxolanosta-8,24-dien-26-oic acid	*Bacillus cereus* *Enterococcus faecalis*	[50]
*Ganoderma pfeifferi*	Ganomycin A	*Bacillus subtilis* *Micrococcus flavus* *Staphylococcus aureus*	[24]
*Pseudoplectania nigrella*	Plectasin	*Bacillus cereus* *Bacillus thuringiensi* *Corynebacterium diphtheriae* *Corynebacterium jeikeium* *Enterococcus faecalis* *Enterococcus faecium* *Staphylococcus aureus* *Staphylococcus epidermidis*	[51,52]
*Clitocybe nebularis*	Nebularine	*Mycobacterium tuberculosis*	[58]
*Pleurotus sajor–caju*	Ribonuclease	*Pseudomonas aeruginosa* *Staphylococcus aureus*	[59]
*Gymnophilus spectabilis*	Hepta-4,6-diyn-3-ol 7-Chloro-hepta-4,6-diyn-3-ol	Gram-positive/Gram-negative	[61]
*Hohenbuehelia grisea*	Pleurotin	Gram-positive bacteria	[62]
*Albatrellus flettii*	Confluentin Grifolin Neogrifolin	*Bacillus cereus* *Enterococcus faecalis*	[50]
*Lentinula edodes*	Oxalic acid	*Bacillus cereus* *Staphylococcus aureus* *Streptococcus faecalis*	[63]
*Cortinarius basirubencens*	Austrocortilutein Austrocortilutein Austrocortirubin Torosachryson	*Staphylococcus aureus*	[66]
*Boletus* spp.	Boletusin Chrysospermin	*Bacillus subtilis* *Corynebacterium lilium* *Staphylococcus aureus*	[55]
*Pycnoporus sanguineus*	Phenoxazin-3-one	*Staphylococcus aureus* *Streptococcus* spp.	[67]
*Ganoderma pfeifferi*	Terpenes	*Escherichia coli* *Proteus mirabilis* *Serratia marcescens*	[24]
*Xylaria intracolarata*	Cloratin A	*Escherichia coli* *Klebsiella pneumonia* *Pseudomonas aeruginosa* *Salmonella enteritidis*	[65]
*Leucopaxillus albissimus*	Chinoline	*Achromobacter xyloxidans* *Acinetobacter baumannii* *Burkholderia cenocepacia* *Burkholderia loccose* *Burkholderia multivorans* *Cytophaga johnsonae* *Pseudomonas aeruginosa*	[67]

**Table 3:** Summary of antibacterial activity of fungal origin extracts.

Species	Extract	Bacteria	References
*Ganoderma lucidum*	Acetone extract Aqueous extract Ethanol extract Methanol extract	*Bacillus subtilis* *Escherichia coli* *Klebsiella pneumoniae* *Pseudomonas aeruginosa* *Salmonella typhimurium* *Staphylococcus aureus*	[74]
*Ganoderma lucidum*	Acetone extract	*Bacillus subtilis* *Klebsiella oxytoca*	[75]
*Hygrophorus* *agathosmus*	Chloroform extract	*Bacillus subtilis* *Enterobacter aerogenes* *Escherichia coli* *Pseudomonas aeruginosa* *Salmonella typhimurium* *Staphylococcus aureus* *Staphylococcus epidermidis*	[76]
*Suillus collitinus*	Dichloromethanol extract	*Bacillus subtilis* *Staphylococcus epidermidis*	[76]
*Hypholoma* *fasciculare*	Methanol extract	*Bacillus cereus* *Bacillus subtilis* *Staphylococcus aureus*	[77]
*Laetiporus sulphureus*	Ethanol extract	*Bacillus cereus* *Bacillus subtilis* *Micrococcus flavus* *Micrococcus luteus*	[78]
*Lentinula edodes*	Chloroform extract Acetate-ethyl extract	*Actinomyces* spp. *Lactobacillus* spp. *Porphyromonas* spp. *Prevotella* spp. *Streptococcus* spp.	[79]
*Leucopaxillus giganteus*	Methanol extract (mycelial cultures)	*Bacillus cereus* *Bacillus subtilis* *Staphylococcus aureus*	[81]
*Navesporus loccose* *Phellinus rimosus*	Methanol extract	*Bacillus subtilis* *Staphylococcus aureus*	[82]
*Pleurotus ostreatus* *Meripilus giganteus*	Ethanol extract	*Sarcina lutea*	[83]
*Trametes versicolor*	Methanol extract	Gram-positive bacteria	[91]
*Grifola frondosa*	Ethanol extracts/polysaccharides	*Bacillus* *cereus*	[84]
*Gloeoporus* *thelephoroides* *Hexagonia hydnoides* *Phellinus* spp.	Acetate-ethyl extract	*Bacillus cereus*	[85]
*Nothopanus* *hygrophanus*	Acetate-ethyl extract	*Listeria monocytogenes* *Staphylococcus aureus*	[85]
*Agaricus bisporus* *Pleurotus sajor*–*caju*	Aqueous extract Ethanol extract Methanol extract Xylene extract	*Enterobacter aerogenes* *Escherichia coli 390* *Escherichia coli 739* *Klebsiella pneumoniae* *Pseudomonas aeruginosa*	[86]
*Hydnum repandum*	Methanol extract	*Pseudomonas aeruginosa*	[87]
*Lepista nuda*	Methanol extract	*Escherichia coli* *Pseudomonas aeruginosa*	[88]
*Suillus collitinus*	Dichloromethane extract	*Bacillus subtilis* *Candida albicans* *Enterobacter aerogenes* *Escherichia coli* *Salmonella typhimurium* *Staphylococcus aureus* *Staphylococcus epidermidis*	[76]
*Hygrophorus* *agathosmus*	Chloroform extract	*Bacillus subtilis* *Enterobacter aerogenes* *Salmonella typhimurium* *Staphylococcus aureus* *Staphylococcus epidermidis*	[89]
*Laetiporus sulphureus*	Ethanol extract Aqueous extract	*Bacillus subtilis* *Bacillus cereus* *Micrococcus luteus* *Micrococcus flavus* *Klebsiella pneumoniea* *Listeria monocytogenes*	[78,90]

**Table 4:** Summary of antifungal activity of compounds of fungal origin.

Species	Chemical Compound/Extract	Fungal Pathogen	Reference
*Trichaptum biforme*	Biforminic acid Biformin	*Aspergillus niger*	[93]
*Penicillium* spp.	Griseofulvin	*Epidermophyton* *Microsporum* *Trichophyton*	[94]
*Agrocybe cylindracea*	Agrocybin	*Fusarium oxysporum* *Mycosphaerella arachidicola*	[96]
*Cordyceps militaris*	Cordymin	*Bipolaris maydis* *Candida albicans* *Mycosphaerella arachidicola* *Rhizoctonia solani*	[97]
*Lactarius rufus*	Rufuslactone	*Alternaria alternata* *Alternaria brassicae* *Botrytis cinerea* *Fusarium graminearum*	[101]
*Flammulina velutipes*	Enokipodins F, G, I	*Aspergillus fumigatus*	[49]
*Tricholoma giganteum*	Trichogin	*Fusarium oxysporum* *Mycosphaerella arachidicola* *Physalospora piricola*	[102]
*Gloeophyllum sepiarium*	Oospolactone	*Alternaria* spp. *Fusarium* spp. *Giberella* spp. *Penicilim* spp. *Aspergillus* spp.	[103]
*Lentinula edodes*	Lentin	*Mycosphaerella*	[104]
*Pleurotus eryngii*	Eryngin	*Fusarium oxysporum* *Mycosphaerella arachidicola*	[107]
*Hypsizigus marmoreus*	Hypsin	*Botrytis cinerea* *Fusarium oxysporum* *Mycosphaerella arachidicola* *Physalospora piricola*	[108]
*Strobilurus tenacellus*	Strobilurins	*Aspergillus panamensis* *Candida albicans* *Paecilomyces varioti* *Penicillium notatum* *Rhodotorula glutinis*	[109]
*Hygrophorus chrysodon*	Chrysotriones A, B	*Fusarium verticillioides*	[105]
*Ganoderma annulare*	5-Ergost-7-en-3-ol 5-Ergosta-7,22-dien-3-ol 5,8-Epidioxy-5,8-ergosta-6,22-die -3-ol Applanoxidic acids A, C, F, G, H	*Microsporum canis* *Trichophyton* *mentagrophytes*	[106]
*Ganoderma lucidum*	Ganodermin	*Botrytis cinerea* *Fusarium oxysporum* *Physalospora paricola*	[110]
*Pleurotus sajor–caju*	Ribonuclease	*Fusarium oxysporum* *Mycosphaerella arachidicola*	[59]
*Crepidotus fulvifibrillosus*	Strobilurins	*Alternaria porri* *Aspergillus ochraceus* *Candida albicans* *Cladosporium cladosporioides* *Curvularia lunata* *Epicoccum purpurascens* *Mucor miehei* *Nematospora coryli* *Neurospora crassa* *Paecilomyces varioti* *Penicillium islandicum* *Penicilium notatum* *Phoma clematidina* *Phytophthora infestans*	[109]

**Table 5:** Summary of antifungal activity of extracts of fungal origin.

Species	Extract	Fungal Pathogen	Reference
*Albatrellus dispansus*	Ethanol extrat	*Erysiphe graminis* *Sclerotinina sclerotiorum* *Fusarium graminearum*	[98]
*Agaricus bisporus*	Aqueous extract Ethanol extract	*Aspergillus flavus*	[111]
*Ophiocordyceps* *sobolifera*	Ethanol extract	*Candida albicans*	[112]
*Agaricus bisporus* *Agaricus bitorquis* *Agaricus sylvicola*	Methanol extract	*Candida albicans* *Candida tropicalis*	[113]
*Hygrophorus* *agathosmus*	Chloroform extract	*Saccharomyces cerevisae*	[76]
*Suillus collitinus*	Dichloromethane extract	*Candida albicans* *Saccharomyces cerevisae*	[76]
*Ganoderma lucidum*	Ethanol extract	*Aspergillus fumigatus* *Aspergillus versicolor* *Aspergillus ochraceus* *Aspergillus niger* *Trichoderma viride* *Penicillium funiculosum* *Penicillium ochrochloron* *Penicillium verrucosum*	[75]
*Laetiporus sulphureus*	Ethanol extract Aqueous-ethanol extract	*Candida albicans* *Aspergillus niger* *Botrytis cinerea* *Fusarium oxysporum,* *Penicillium gladioli* *Sclerotinia sclerotiorum*	[78]
*Lactarius camphoratus*	Methanol extract	*Candida albicans*	[116]
*Lentinula edodes*	Chloroform extract	*Candida albicans*	[63]
*Lepista nuda*	Methanol extract	*Candida albicans*	[88]
*Trametes versicolor*	Methanol extract	*Aspergillus fumigatus*	[91]

**Table 6:** Summary of antiviral activity of compounds and extracts of fungal origin.

Species	Chemical Compound/Extract	Virus Type	Reference
*Tricholoma giganteum*	Trichogin	HIV-1	[102]
*Ganoderma lucidum*	Ganoderiol Ganodermanontriol Ganodermic acid	HIV-1	[110]
*Ganoderma pfeiferi*	Ganodermadiol	H1N1	[118]
*Inonotus hispidus*	Phenolic compounds	H1N1and B	[119]
*Trametes versicolor*	PSK complex	Cytomegalovirus HIV	[120]
*Agaricus brasiliensis*	Aqueous extracts	HSV	[126]
*Rozites caperata*	Aqueous extracts	HSV	[122]
*Ganoderma pfeifferi*	Aqueous extracts	HSV	[118]
*Pleurotus pulmonarius*	Ethanol extract	(H1N1pdm)	[123]
*Cordyceps militaris*	Polyssaccharide acidic fraction	H1N1	[124]
*Laetiporus sulphureus*	Aqueosus-methanol extract	HIV	[136]
*Agaricus brasiliensis*	Polysaccharides	PV-1	[121]
*Porodaedalea pini*	EP-AV1 polysaccaride EP-AV2 polysaccaride	HSV-1 CVB3	[137]
*Phellinus baumii*	Hispidin Hypholomine B Inoscavin A Davallialactone Phelligridin D	H1N1, H5N1, H3N2	[128]
*Agaricus bisporus* *Pleurotus ostreatus*	Laccase enzyme Tyrosinase enzyme	HCV	[129]
*Grifola frondosa*	Β-glucan	HBV	[131]
*Lentinula edodes*	Lentinan	IHNV	[132]
*Inonotus obliquus*	Polysaccharide fraction Aqueous extract	COVID-19 HCV	[133] [135]

## Data Availability

Data sharing is not applicable.
